# Recent Applications of In Silico Approaches for Studying Receptor Mutations Associated with Human Pathologies

**DOI:** 10.3390/molecules29225349

**Published:** 2024-11-13

**Authors:** Matteo Pappalardo, Federica Maria Sipala, Milena Cristina Nicolosi, Salvatore Guccione, Simone Ronsisvalle

**Affiliations:** 1 Department of Drug and Health Sciences, University of Catania, Viale A. Doria 6, 95125 Catania, Italy; mpappala@unict.it (M.P.); federica.sipala@phd.unict.it (F.M.S.); milena.nicolosi@phd.unict.it (M.C.N.); simone.ronsisvalle@unict.it (S.R.); 2 Department of Chemical Science, University of Catania, Viale A. Doria 6, 95125 Catania, Italy

**Keywords:** in silico approaches, mutations, receptors, molecular modeling, docking, molecular dynamics

## Abstract

In recent years, the advent of computational techniques to predict the potential activity of a drug interacting with a receptor or to predict the structure of unidentified proteins with aberrant characteristics has significantly impacted the field of drug design. We provide a comprehensive review of the current state of in silico approaches and software for investigating the effects of receptor mutations associated with human diseases, focusing on both frequent and rare mutations. The reported techniques include virtual screening, homology modeling, threading, docking, and molecular dynamics. This review clearly shows that it is common for successful studies to integrate different techniques in drug design, with docking and molecular dynamics being the most frequently used techniques. This trend reflects the current emphasis on developing novel therapies for diseases resulting from receptor mutations with the recently discovered AlphaFold algorithm as the driving force.

## 1. Introduction

The field of computational chemistry, particularly in drug design, has become increasingly important for the practical application of predictive modeling. The application of computational methods, in particular molecular modeling, has facilitated the prediction and simulation of molecular and protein behaviors, thereby enabling a deeper understanding and evaluation of the interactions between an experimental molecule and the receptor binding site [1,2]. In addition, computational techniques have been employed to predict and determine the three-dimensional structure of proteins. Recently, new methods such as AlphaFold have been developed to predict and discern the three-dimensional structure of proteins with atomic precision [3]. This innovation has revolutionized the discovery and identification of new lead compounds. Understanding the mechanism by which a ligand interacts with its biological target is essential to comprehend its functional properties. Biological recognition of a molecule occurs through a system of attractive and repulsive interactions between the ligand and biological target [4]. Computational methods are commonly employed for three primary purposes: identification of the active site of an enzyme or the binding site in a receptor, the exploration of novel, selective bioactive ligands for drug targets, and modification of existing drugs to enhance their efficacy and diminish side effects or to redirect them to new drug targets [5,6]. For the proper execution of various computational structure-based methods, such as docking and molecular dynamics, it is necessary to know the three-dimensional structure of the protein target. The number of protein structures has increased over the last 30 years, supported by the increase in X-ray crystallographic techniques, including nuclear magnetic resonance (NMR) and cryoEM, used to accurately and efficiently obtain crystal structures [7,8]. The Protein Data Bank (PDB) is a database of protein structures obtained using various techniques. Currently, the PDB contains over 225,681 structures of proteins, including those of mutated receptors [9,10]. The wild type and mutated structures of the proteins involved in diseases are crucial for the study of several disorders. Therefore, in silico mutagenesis is essential to identify the potentially relevant amino acids involved in numerous pathologies. The structural information obtained from molecular modeling helps in understanding whether the mutation in a specific amino acid is relevant for a disease. These results will be useful for further in vitro mutagenesis studies [11]. This enables the development of targeted therapies and drugs that can specifically target mutated receptors to treat or manage certain diseases. Receptor mutations are derived from genetic changes or variations in DNA sequences that affect the structure and function of cellular receptors. These mutations can occur spontaneously during DNA replication and can be induced by environmental factors, such as radiation or chemicals, or inherited from parents [12]. Mutations can have different effects on receptor function [13]. They have the ability to alter the capacity of the receptor to bind with its ligand, impact its activity level, or disrupt signal transmission. For example, amino acid mutations in G-coupled receptors can have two primary effects on protein signaling: disrupting the signal and rendering the receptor inactive or altering the signal and changing the activation pathway or outcome. Receptor mutations resulting in disrupted signals are referred to as loss-of-function (LOF) mutations [14]. Conversely, gain-of-function (GOF) mutations can result in protein overexpression or overwork of the protein [15]. These mutations can lead to pathological conditions or alterations in cellular responses [16]. In fact, pathological conditions can be caused by mutations that interfere with the normal functioning of genes involved in critical cellular processes, including cell growth, division, and DNA repair [17].

This review intends to demonstrate the application of computational techniques in examining receptor mutations associated with various pathologies. We conducted a search of the PubMed database using specific keywords, including “mutant”, “protein or receptor”, and “molecular modeling or computational chemistry”. Our initial search was conducted on the titles and abstracts of articles, excluding those published before 2020. Subsequently, a full-text search was performed to exclude articles that did not meet our specific purposes. This review emphasizes that authors predominantly employed well-established molecular modeling methods, which are outlined in detail in the following sections.

## 2. Overview of Computational Methods 

### 2.1. Homology Modeling

Predicting the tertiary structure of a protein is a crucial aspect of structure-based drug design, which can ultimately lead to the development of more effective drugs. The tertiary structure of a protein can be predicted using various techniques that depend on the availability of the tertiary structure of a similar protein. Homology modeling techniques can be employed when structures are accessible. In cases where these methods are not applicable, ab initio, threading, and AlphaFold methods can serve as suitable alternatives.

**Figure 1:**
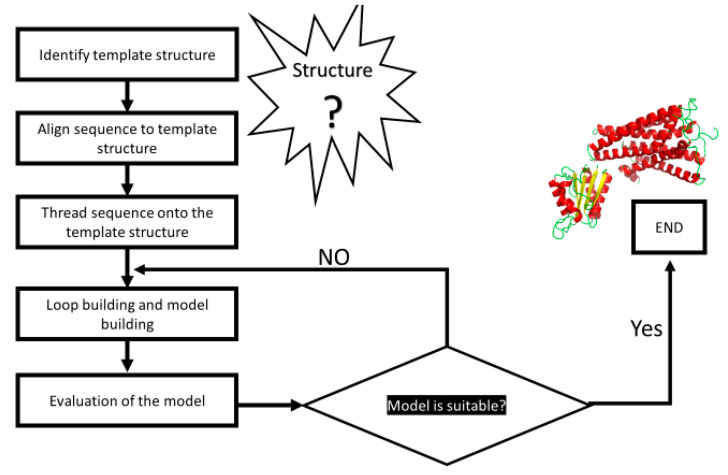
Generic workflow for homology modeling.

Homology modeling aims to construct a three-dimensional (3D) model for a protein of unknown structure based on its sequence similarity with proteins of known structure, obtained using common crystallographic techniques such as X-ray crystallography or NMR spectroscopy; a workflow is reported on Figure 1 [18]. The amino acid sequence is used to predict this structure. Initially, the key step is to determine all protein structures that correspond to the target sequence and then choose the most appropriate one to serve as a reference. These structures can be found in the PDB, scop, DALI, and CATH [9,19,20,21,22]. Protein model templates with more than 50% sequence identity are generally highly accurate. In order to obtain a realistic model, the percentage of similarity of structures must be approximately 30–50%. If there is a high percentage of primary sequence similarity, it can usually be assumed that there is also good structural similarity [23]. The process of homology or comparative modeling of proteins consists of several steps [24]. Firstly, a basic structure is selected from the PDB, which is used as a template for construction. The most popular server is the Basic Local Alignment Search Tool (BLAST) [25]. However, BLAST is not able to find templates in cases where the percentage of similarity is less than 30%. In situations like these, Hidden Markov Models (HMMs) and position-specific iterated BLASTSs (psi-BLASTs) are employed as alternative alignment models. Sometimes, the transitive property can be used in cases in which it is not possible to find a template structure. For example, if the structures of proteins A and B are not structurally similar, but they are both similar to protein C, it is possible to say that A and B are similar. Then, the sequence alignment of the target and template proteins is a highly specialized process that requires great care, as errors cannot be corrected once made. However, when the compatibility percentage is greater than 50%, this method is highly precise. The third step is model building, which involves four different approaches: rigid body assembly, segment matching, spatial restraint, and artificial evolution. This technique involves observing the sequence to determine which parts of a particular class of proteins are constant and to understand which parts are changing. The rigid-body assembly method collects the rigid part of the protein using SWISS MODEL [26]. Segment matching compares the database structure based on the sequence identity, geometry, and energy using the SegMod/ENCAD program. Spatial restraint is another method that depends on the restraints of the template and can be performed using MODELLER [27]. Artificial evolution depends on rigid-body assembly and stepwise template evolutionary mutations and can be performed using NEST [28]. Finally, the model is evaluated. This step is crucial for interpreting the final 3D structure because errors may be accidentally introduced and propagated. The protein model can be evaluated as a whole and in individual regions [29].

Ab initio modeling and threading are two different strategies employed for predicting protein structures when experimental data is unavailable. Ab initio modeling is a technique employed to predict the three-dimensional structure of a protein using only its amino acid sequence, without requiring templates of known structures. This method relies on an accurate energy function that enables a conformational search, resulting in several potential protein conformations. Ultimately, a probable model is chosen from the generated options [30]. The accuracy of predictors of inter-residue contact maps has been substantially enhanced over the years, thanks to the advancement in deep-learning techniques and the expansion of protein sequence databases. This has resulted in more precise predictions of the three-dimensional structure of proteins. Contact maps encode information about interatomic interactions, which can be utilized for highly precise protein structure prediction through contact map threading, even for query proteins that are unsuitable for direct homology modeling. Consequently, contact-assisted threading has attracted substantial research interest [31]. An innovative approach for determining protein structure is AlphaFold, which is based on a sophisticated Artificial Intelligence algorithm that uses deep neural networks and machine learning to predict distances between amino acid residues and local torsion angles, which is then integrated to determine the final structure of the protein. The neural networks are trained on databases of protein structures. AlphaFold version 3 of which has recently been delivered has proven to be a prediction software with a high level of accuracy, allowing reliable models to be obtained [3,32,33,34,35]. 

### 2.2. Virtual Screening and Docking

Docking is a multistep process employed to predict and understand the structural and energetic features underlying the interaction between bioactive compounds and biological targets [36]. The objective is to identify the most suitable binding arrangement of a ligand within a protein to form a stable complex. During the docking process, various conformations of the molecules are generated and evaluated to determine the most energetically favorable or biologically relevant binding mode. Frequently, numerous approximations must be incorporated into the procedure, both in the force field and in the conformation search, to facilitate docking with a reasonable level of computational effort. These approximations include the use of simplified force fields, restriction of the search space, and limitations of the conformational flexibility of the ligand and/or target [37]. The result of the docking calculation is a large number of binding poses, which are evaluated using scoring functions. Scoring functions are mathematical algorithms that assess and assign numerical values to various structural arrangements or poses of molecules within a complex. They aim to estimate the binding affinity or energy between the molecule and the protein, assigning a numerical value to each conformation [38,39]. Although virtual screening and docking enable a rapid and efficient prediction of the interactions of a ligand with its biological target, these methods consider simplified models that do not take into account the flexibility of the protein and do not account for movements over time. Ensemble docking approaches that use multiple protein conformations can help to overcome the flexibility problem.

Virtual Screening (VS) is a computational technique that allows for the identification of potential drug candidates from compound libraries or databases. It is employed in drug design and discovery to reduce the time and cost associated with traditional high-throughput screening methods. VS techniques employ algorithms to predict the binding of a compound to a selected receptor and to predict which compounds are most likely to have the desired biological activity [40,41]. Virtual screening in drug discovery has become an essential tool to assist hit identification, given the extensive timeframe of approximately 10–15 years required for the development and approval of new pharmaceuticals [42]. In silico techniques are powerful tools also in drug repurposing, offering a cost-effective and efficient means to identify new therapeutic uses for existing drugs. They complement traditional experimental approaches and have the potential to accelerate the drug discovery process significantly. This method consists of taking a drug that has already been approved by regulatory agencies and testing its activity on other proteins or receptors to speed up the process [43]. Drug repurposing can also involve the recovery of abandoned molecules, a process known as “drug rescue”. This approach involves the application of computational methods to study molecules that were previously developed but abandoned for a variety of reasons, such as lack of efficacy for the original purpose, safety issues, and regulatory difficulties. Drug rescuing is important to lower the costs and the time associated with new drug discovery and development [44,45]. Two main methods of virtual screening have been recognized based on the available structural information. The structure-based virtual screening (SBVS) method relies on the three-dimensional structure of the target protein, which is often obtained experimentally through X-ray crystallography, NMR, electron cryomicroscopy, or in silico by homology modeling. Algorithms are used to predict how potential drug compounds interact with a target protein based on their shape, electrostatic properties, and other factors. Several poses of the ligand in relation to the protein are generated and then evaluated based on their binding affinities through a scoring function. To enable the screening of large chemical libraries, docking programs make several approximations. One of these is to treat the ligand as flexible and disregard the flexibility of the protein. In addition, in the scoring function, some contributions of the solvent or flexibility of the ligand are approximated or neglected [46,47]. Ligand-based virtual screening (LBVS) is based on the properties of known active or inactive compounds for a selected target. Computational methods, such as pharmacophore modeling, quantitative structure-activity relationship (QSAR) analysis, and similarity searching, are used to identify compounds with structural features or biological activity similar to those of known ligands. Pharmacophoric models are based on the molecular properties required for the identification of a ligand by a biological target, and they determine the similarity value of the proposed ligand. Among the software employed to create pharmacophore models, there are Phase from the Schrödinger suite, Molecular Operating Environment (MOE), LigandScout, and others [48,49]. LigandScout is a software that exploits the structural data of a protein–ligand complex to develop three-dimensional pharmacophoric models [50]. 3D-QSAR methods investigate the relationship between biological activity and interaction areas. The first 3D-QSAR technique to consider interaction areas was Comparative Molecular Field Analysis (CoMFA). This method examines electrostatic and steric-type interactions as the primary interaction forces [23]. Software used for virtual screening includes GLIDE, Dock BLAST, DOVIS 2.0, AutoDock Vina 1.2.5, GNINA 1.0, Vina LC, POAP, and DOCKTITE [51,52,53,54,55,56,57]. In the study of novel ligands associated with wild-type and mutated proteins, virtual screening has been used to skim libraries of compounds and identify those with the best characteristics, choosing from those with the best scoring values and interactions with the receptor [58]. 

### 2.3. Molecular Dynamics

Molecular Dynamics (MDs) is a computational method that uses experimental structural data to predict the possible conformations of molecular systems over time. These simulations reveal conformational changes, ligand binding, and protein folding, showing the positions of all atoms with femtosecond temporal resolution. Such simulations can also predict how biomolecules respond to perturbations such as mutation, phosphorylation, protonation, or ligation [59]. The interactions between the atoms are described by the potential energy function, commonly referred to as the “force field”. It consists of bonded terms between covalently bonded atoms (i.e., bonds, angles, and torsions) and unbonded terms (i.e., van der Waals and electrostatic interactions) [60]. The force exerted on each atom by all the other atoms in a biomolecular system can be calculated from the positions of all the atoms. The spatial position of each atom can then be predicted as a function of time using Newton’s law of motion. In particular, the forces on each atom are repeatedly calculated and then used to update the position and velocity of each atom.

The Newton equation is as follows:

$$f_{i}=m_{i}a_{i}=m\cdot \frac{d^{2}r_{i}}{dt^{2}}=f_{i}=-\frac{\partial }{\partial r_{i}}U\left(r_{1},r_{2}\ldots r_{N}\right)$$

where m_i_ is the mass of atom i, a_i_ is the acceleration of atom i, and f_i_ is the force acting on atom i given by the partial spatial derivative of the potential energy function V, which is dependent on the position r_N_ = (r_1_, r_2_, ..., r_N_) [60,61,62]. In particular, the forces on each atom are repeatedly calculated and then used to update the position and velocity of each atom. The resulting trajectory is essentially a three-dimensional movie that describes the atomic-level configuration of the system at every point during the simulated time interval. The most established computational tools available for classical MD simulations are the software suites GROMACS, Nanoscale Molecular Dynamics (NAMD), CHARMM, AMBER, Desmond from the Schrödinger suite, and Biovia Discovery Studio, which can be used in all computer classes: personal computer supercomputers (using multiprocessors) and calculations can be accelerated by GPU (NVIDIA CUDA) [63,64,65,66,67,68,69,70,71]. Coordinates of atoms and appropriate values are estimated through a fixed time step and an integration step. The outputs of the MD simulations are trajectories representing snapshots of the evolution of the system and appropriate values of time, energy (e.g., van der Waals), applied force, and temperature of the system, and are essentially a three-dimensional movie [72]. The root mean square deviation (RMSD) is employed to assess the variation in the structural configuration of a protein from its initial state and during the entire simulation [73]. Molecular dynamics has facilitated drug design by enabling the consideration of flexibility in both ligands and receptors, as well as the accurate temporal evolution of the system. However, conducting these simulations is highly time-consuming and requires substantial computational resources.

### 2.4. Electron Density

Kang et al. introduced a novel approach to describe non-covalent intermolecular interactions (NCIs) by reducing the electronic density gradient (RDG) using electronic density (ED) and its first derivative based on experimental PDB data. This method has been successfully applied in various fields, from small-molecule database creation to receptor studies, and is expected to be increasingly used, especially in rare diseases [74]. The electron density approach has been applied to several studies for the creation of new databases of small molecules or for receptor studies and more applications are expected in the field of rare diseases [75].

### 2.5. Free Energy Calculations

Several molecular modeling techniques combine molecular mechanics with either the Poisson–Boltzmann or generalized Born and surface area continuum solvation methods to determine the binding free energy of a ligand–target complex. These methods are MM/PBSA and MM/GBSA. These methods estimate the free energy of binding between a ligand and a protein, considering both the molecular interactions and the solvent contribution. MM/GBSA combines molecular mechanics (MM) with the Generalized Born (GB) model to estimate the electrostatic energy of solvation and the Surface Area (SA) model to estimate the apolar contribution. In particular, MM considers covalent and non-covalent interactions between atoms, GB calculation considers the effect of the solvent on the electrostatic interactions, and the SA model considers the non-polar interactions between the molecule and the solvent. The MM/PBSA methods use the Poisson–Boltzmann (PB) approach to calculate the effect of the solvent on the electrostatic interactions. This method is more accurate and therefore requires a longer computing time [76,77]. Another widely used in silico method for calculating the free energy of a ligand–target complex is free energy perturbation (FEP) calculation. In particular, this method uses statistical mechanics to calculate the energy differences between several compounds binding a single protein. For this reason, FEP calculations are employed subsequent to virtual screening calculations or in the context of in silico mutagenesis to investigate the effects of a mutated amino acid [78].

## 3. Overview of Applications of Computational Methods

Computational techniques are significant in studying mutations of several receptor types involved in various pathologies [79,80]. Considerable advancements have been achieved using molecular modeling for the investigation of guanine nucleotide-binding protein (G protein)-coupled receptors, involved in the majority of the activation pathways in the human body. Mutations in these receptors are associated with several diseases as a consequence of their high prevalence in every body district [81]. Another crucial family of receptors, particularly involved in cancer, are tyrosine kinase receptors. Their structure, which includes an extracellular component for binding ligands and a tyrosine kinase domain, enables these receptors to contribute to transcriptional pathways involved in cellular growth and differentiation, therefore rendering them important in cancer. 

### 3.1. G Protein-Coupled Receptors (GPCRs)

G protein-coupled receptors include a transmembrane region, consisting of seven helical segments, an extracellular region important for ligand binding, and an intracellular region that interacts with the G protein. The activation of these receptors by a ligand activates various signaling pathways which are dependent on the type of the G protein involved. Due to the presence of different types of G proteins and therefore different signaling mechanisms, these receptors are implicated in numerous physiological processes. As a result, mutations of GPCRs are involved in various pathological conditions, including cancer, pain, inflammation, movement disorders, and ocular disease [82].

#### 3.1.1. GPCRs and Cancer

An important GPCR involved in cancer is the follicle-stimulating hormone (FSH) associated with ovarian cancer. Zariñán et al. (2021) studied mutations in these receptors using docking and molecular dynamics simulations [83]. Loss of function of this receptor is a rare cause of primary ovarian failure, due to mutations I423T and D408Y inside the receptor. To study the effect of mutations, molecular dynamics simulations were performed with a duration of 100 ns for the wild type of receptor and for WT FSHR (N680), WT FSHR variant (S680), and FSHR I423T (S680) mutants. The FSHR I423T/N680S mutant phenotype was generated, mutating the amino acid residues I423 and N680 to threonine and serine, respectively. The FSHR model was obtained using GPCR-I-TASSER [70,84].

Among the GPCRs of interest for tumor research, adenosine receptors (AR) have been observed to be upregulated in various tumor cells. There are three receptor types, each exhibiting a distinct function in tumor modulation [85]. Specifically, the human A2A adenosine receptor (A2AAR) receptor subtype is known to inhibit the immune response to tumors. The activation of these receptors by specific ligands, agonists, or antagonists modulates tumor progression. Conversely, the A2BAR receptor subtype is implicated in the expression of angiogenic factors [86]. De Filippo et al. (2020) manipulated the extracellular loop 2 (ECL2) of the A2AAR to resemble that of the A2B adenosine receptor (A2BAR), which resulted in a mutant receptor [87]. This modification was based on the evidence that ECL2 is crucial for ligand recognition and receptor activation in adrenergic receptors. The resulting chimeric A2A(ECL2-A2B) AR was investigated in radioligand binding and cyclic adenosine monophosphate (cAMP) accumulation assays and compared to the wild-type A2AAR. The homology model of the human A2A(ECL2-A2B) mutant was obtained by superposing the intermediate active state A2BAR model obtained from adenosine to the corresponding A2AAR template (PDB ID: 2YDO) [88,89]. The ECL2 residues were inserted between Asn144ECL2 and Ala165ECL2. These new backbone bonds were minimized using MOE software, and the Amber99 force field was employed [48,67,68]. Ligand–receptor interaction energies were calculated by extrapolating the non-bonded energy interaction term of the CHARMM27 forcefield using NAMD [64,65,66]. All molecular dynamics simulations were performed using the ACEMD software [90]. In this case, De Filippo predicted a new meta-binding site with high affinity for adenosine, but not for NECA, which may contribute to future drug design. To investigate its role in cancer, Wang et al. (2020) observed the adenosine A2 receptor activity [91]. When activated by high levels of endogenous ligands such as adenosine, it inhibits the immune response to tumor progression. However, this did not occur when the receptor was mutated. Fifteen point mutations in the adenosine A2B receptor have been identified in cancer patients; three mutations were in the extracellular loop (EL), two in the intracellular loop (IL), six in the 7-transmembrane domain, and four at the C-terminus of the adenosine A2B receptor. They used GPCR-ModSim to perform homology modeling against a set of crystallized GPCRs that were divided into inactive, active-like, and fully active. The template used was the adenosine A2A receptor using the structures deposited in the PDB with code 3EML for the inactive model, code 2YDV for the active-like structure, and code 5G53 for the fully active structure [88,92,93]. Homology models were generated and sorted by the server using the DOPHR scoring function after adjusting the extracellular loop 2 (ECL2) region using MODELLER [27,94]. This study for the first time tried to elucidate the nature of adenosine A2B receptor cancer mutations, with a future perspective of insights into mutant receptor function in cancer.

#### 3.1.2. GPCRs and Pain

In the field of pain, GABA receptors have been proved to have an important role in pain signal transmission, in both fast and slow synaptic transmission. Bony et al. (2022) investigated the effect of α-conotoxins on GABABR receptors to study their binding as allosteric modulators of these receptors. They employed computational methods to evaluate how mutations at the VFT domain interface of GABABR subunits affect the reduction in baclofen-sensitive IBa inhibition by pain-relieving α-conotoxins [95]. Discovery Studio Visualizer was used to modify the parent conotoxins to create the analogue RgIA4 [69,96,97,98,99]. Molecular docking calculations were performed using AutoDock Vina [100]. The most favorable docking positions were selected to perform MD simulations using GROMACS 2019 and the CHARMM36m force field [63,101]. Each complex was subjected to 1000 ns of molecular dynamics and the results of MD simulations were analyzed using VMD [102]. In this work, the authors adopted a docking/MD approach to identify a novel allosteric binding site for the analgesic α-conotoxins at the interface between the VFT domains of the GABABR subunits, allowing future research in the area.

Cannabinoid receptors are G protein-coupled receptors involved in several physiological events. In particular, there are two known receptor subtypes: cannabinoid receptor 1 (CB1) and cannabinoid receptor 2 (CB2). CB1 receptors are mainly located in the central nervous system (CNS), gastrointestinal tract, cardiac system, and liver. CB1 receptors participate in certain central physiological activities, such as pain, and peripherals, such as energy metabolism and cardiovascular function. CB2 receptors are also expressed in the immune system [103]. The research conducted by Casajuana-Martin et al. (2022) focused on molecular dynamics simulations and site-directed mutagenesis to study the binding of the agonist JWH-133 to the cannabinoid CB2 receptor (CB2R) [104]. GROMACS 2016 was employed to perform the molecular dynamics simulations (five replicas of 1 μs) using the force fields implemented in Amber [63,67,68]. Bonds involving hydrogen atoms were frozen using the LINCS algorithm, trajectory analysis was performed using MD Analysis, and the membrane stability was monitored using FATSLiM [105]. PyMOL and VMD were used for visualization and image plotting, respectively [102,106]. This study demonstrated key interactions with amino acids at the entrance of the binding site and that Ala2827.36Phe mutation blocks the entry of the ligand, which exhibits lipophilic characteristics and spreads through the lipid bilayer by obstructing its interaction with the ligand-binding pocket of the receptor. del Torrent et al. (2023) used a combination of MD simulations and site-directed mutagenesis to identify the residues at position 6.51 in the CB1R and CB2R receptors as crucial components in the selective recognition of Δ9-tetrahydrocannabinol alkyl chains [107]. Specifically, they proposed that Leu in CB1R and Val in CB2R play a critical role in this process. They studied the effect of THC on a mutant CB2RV6.51L receptor, which is as efficient as the wild-type CB1R. MD simulations of these models were performed with GROMACS 2018.5.31 using the General Amber Force Field (GAFF2) [63,67,68]. The molecular systems were subjected to 5000 steps of energy minimization using the steepest descent algorithm and PME electrostatics with the Verlet cutoff scheme. This was followed by a 25 ns equilibration protocol consisting of six steps. Trajectory analysis was performed using MD Analysis, visualization and image rendering was performed using PyMOL36 and VMD, and graphing was performed using the Seaborn package [102,106,108]. This study, coupling experimental techniques with refined MD calculation, was able to elucidate the initial mechanism of activation of GPCRs. 

#### 3.1.3. GPCRs Involved in Other Diseases

In a study conducted by Chan et al. (2020), melatonin MT1 and MT2 receptors were found to be promising pharmacological targets for the treatment of various disorders, including Alzheimer’s disease, Parkinson’s disease, and depression [109]. Molecular docking was employed to study the amino acids involved in the interactions with the M2-selective isoquinolone ligands. The authors constructed the homology model of MT1 and MT2 receptors using SWISS MODEL using the MT2 receptor (PDB code 6ME6) or human β2-adrenergic receptor (PDB code 3P0G) as templates [26,110,111]. Docking calculations were performed using AutoDock software; specifically, AutoDock Tools were employed to obtain the 3D structures of the ligands, add the Gasteiger charges, set rotatable bonds, and enable the rotation of the torsions [100]. Grid maps were generated using Autogrid. The potential binding sites of the ligand were identified using SITEHOUND and FunFOLD software [112,113]. Specifically, SITEHOUND detects areas with advantageous non-bonded interactions using a chemical probe, which are potential binding sites. The development of molecular interaction field maps is initiated by the algorithm, followed by the identification of the protein probe with the lowest interaction energy as the potential binding site [112]. FunFOLD uses structural similarity within a particular protein family to predict the location of a binding site [113]. The present method utilized a clustering approach to identify the most prevalent clusters of ligand-bound proteins. To achieve this, it combines multiple structures of ligand-bound proteins and subsequently selects the cluster with the highest number of ligands. The area identified as the putative binding pocket is a region within the protein family where ligands typically bind. Nineteen amino acid residues are in the upper portion of the protein and within the extracellular loops, where they interact with the ligands. Of these, seven residues (Asn175, His208, Trp264, Asn268, Gly271, Tyr294, and Tyr298) were identified as crucial for the interaction with the ligand and were chosen by the authors for in vitro mutagenesis. In the end, this complex provides evidence for MT2-selectivity on a structural basis considering a panel of isoquinolinone derivatives, opening up the design of new drugs.

The I212F mutation in the D2 dopamine receptor has been linked to various movement disorders. This mutation is associated with increased receptor activity compared to that of the wild-type receptor. To investigate the consequences of this genetic mutation, Rodriguez-Contreras et al. (2021) performed both in vitro and in vivo experiments and employed molecular dynamics simulations to explore modifications in receptor activation resulting from this receptor [114]. The authors conducted a homology modeling approach using YASARA Structure software to structure the D2 receptor in its inactive state on various receptor structures, including the β2-adrenoceptor, adenosine receptor A2A, M2 muscarinic receptor, bovine rhodopsin, and the D2 receptor in its inactive state [115]. The same technique was used to model the D2 receptor in its active state using the active state structures of the M2 receptor, β2-adrenoceptor, A2A receptor, CB1 receptor, μ-opioid receptor, and rhodopsin. The models were optimized using backbone-dependent probabilities, force fields, and hydrogen bonds. The stereochemical properties of the models were evaluated using the PROCHECK module on the PDB server. The VERIFY3D server was employed to assess the compatibility of three-dimensional (3D) models with their corresponding primary amino acid sequences [116]. Molecular dynamics simulations for a duration of 15 ns were conducted using the YASARA software in NPT mode, with the force fields implemented in AMBER14 [67,68]. The water model used in this study was the TIP3 model. Experiments were carried out at a 0.9% NaCl concentration by mass, pH of 7.4, and temperature of 298 K at atmospheric pressure. Molecular modeling studies have demonstrated that the I212F mutation causes an ionic block between two amino acids, TM3 and TM6. This ionic block maintained the unbound receptor in an inactive conformation. However, this block is disrupted in the presence of an agonist, resulting in the receptor adopting an active conformation and constitutive activation. Molecular dynamics simulations showed that Phe212 in the mutated receptor is located very close to Ser129, establishing an interaction that provides the energy required to separate the ion block. This results in the formation of constitutively active receptors. All these studies provide evidence that the possible pathogenic variant in a specific brain region may depend on GRK expression and the nature of the G protein.

Picarazzi et al. (2022) investigated clinically relevant rhodopsin mutations at the P347 site within the VAPA-COOH motif using MD simulations and compared them with the WT system [117]. The crystal structure of full-length bovine rhodopsin (PDB-ID: 1U19) was used as a template for homology modeling and MD refinement because of the lack of structural information regarding full-length human rhodopsin, as other PDB structures lack the C-terminal tail [118]. Homology modeling was performed using FASTA alignment, which proved to be extremely reliable with 93.4% homology. The structures of WT human rhodopsin and mutants were generated using Schrödinger’s Prime software [119]. Subsequently, MD simulations were performed using the ff14SB force field. To prepare the system, WT and mutated proteins were incorporated into the membrane construct CHARMM-GUI into a 1,2-dioleoyl-sn-gly-cero-3-phosphocholine (DOPC) membrane and a TIP3P water box, and the system was neutralized by adding Na+ [66]. The decapeptides were prepared in the same manner as for the full-length systems. Then, they were solvated in a straight box containing TIP3P water molecules. Subsequently, 250 ns unrestricted MD simulations were performed.

### 3.2. Tyrosine Kinase Receptors

Tyrosine kinase receptors are transmembrane receptors characterized by an extracellular ligand binding site and a catalytic tyrosine kinase site. These receptors are distributed across various tissues, and their functions include cellular differentiation, proliferation, survival, and metabolism [120]. Their tyrosine kinase activity is initiated after ligand binding, leading to phosphorylation of target proteins and the subsequent activation of a signaling cascade that ends in gene transcription. Due to their involvement in cell differentiation, proliferation, and survival, protein mutations resulting in disfunction can lead to various types of cancers [121]. Tyrosine kinase receptors are classified into 20 families. Among these, the most significant for their involvement in tumor progression are the epidermal growth factor receptor (EGFR) and neuregulin (ErbB1) EGFR/ErbB receptors. Mutations in EGFR have been observed as oncogenic drivers in several types of tumors, including in non-small cell lung cancer (NSCLC) and glioblastomas [122]. Mutations in EGFRs have been observed in the extracellular domain of glioblastoma cells, such as R108K, T263P, A289V/D/T, and G598V. Mutations in the EGFR tyrosine kinase domain exert several effects on EGFR activation. The L858R mutation results in a constitutively active receptor with high phosphorylation [123]. The established treatments for tumors caused by EGFR mutations are tyrosine kinase inhibitors. However, this receptor is extensively studied due to drug resistance phenomena. Multiple studies have demonstrated that resistance is attributable to mutations in the receptor, such as T790M and C797S [124]. In order to study the impact of these mutations, and to design new tyrosine kinase inhibitors to overcome the occurrence of resistance, several studies have employed molecular modeling techniques. The study conducted by Akher et al. (2020) examined kinase inhibitor fluorinated derivatives targeting the EGFR in carcinomas [125]. Studies were conducted focusing on WT EGFR and the L858R/T790M/C797S mutated variant. MD simulations and thermodynamic analyses were performed to explore the interactions between difluorinated and non-fluorinated inhibitors with WT and mutated EGFRs. The crystal structure of the EGFR with the L858R/T790M mutation was obtained from the PDB. The co-crystallized structures were removed using MODELLER software, while UCSF Chimera was utilized to perform the C797S residue mutation; consequently, molecular docking was conducted. MD simulations were performed for 300 ns, and the trajectories were analyzed using CPPTRAJ. The binding free energies of the complexes were calculated by the MM/GBSA method. The results showed that the stability of the complex increased for each additional fluorine substituent and that the substitution in the meta position leads to significant conformational changes between the two complexes. This observation highlights the role of fluorides in enhancing the binding affinity. Jingwen et al. (2020) employed computational techniques to study the binding of a series of 5-methylpyrimidine-pyridinone derivatives for EGFR and EGFR mutants [126]. The structure of the receptor was retrieved from the PDB (PDB code 5XDK) [126,127]. Missing residues were added by the SWISS MODEL web server, the L858R and C797S mutations were generated using Discovery Studio 4.0, and docking was simulated using AutoDock Vina software [20,39]. GROMACS 4.5.2 software was used for MD simulations [63]. Protein parameter files were generated using Amber 14.0, using leap.gaff force field [67,68]. This computational study provides crucial insights that will contribute to the creation of novel, highly selective inhibitors targeting oncogenic EGFR. Shaheen et al. (2020) employed molecular docking to study the binding mode and interactions between a series of hexahydroquinoline and fused quinoline derivatives and the EGFR [128]. Particularly, new series of oftetrahydropyrimidoquinolines 5a-f, hexahydropyrimidoquinolines 6a,b, and tetrahydro [1,2,4] triazolopyrimidoquinolines 9a,b were designed, synthetized, and evaluated. The authors employed the MOE software [48]. To study the different binding modes between wild-type and mutated receptors, the authors used the crystal structures of EGFRWT, EGFRL858R, EGFRT790M, and JAK3. Conformational analysis of compound 7d was performed using the MMFF94 force field, and the root mean square (RMS) gradient was 0.01 kcal/mol Å. Compound 7d was aligned using the flexible alignment tool of the program, with an energy cutoff of 15 kcal/mol and an RMSD tolerance of 0.5. Alignment was performed to ensure optimal results. The outcomes of the molecular docking computation for compound 7d were in agreement with the findings of the in vitro experiments, indicating its potential as a promising ligand for EGFRL858R and EGFRT790M mutant receptors. The physicochemical, pharmacokinetic, and toxicological properties of the studied compounds were predicted by the authors. Lipinski’s rule and Veber’s parameters were analyzed with the assistance of Molinspiration software [129]. ADME properties were predicted using PreADMET software [130,131]. In particular, blood–brain barrier penetration, plasma protein binding, and intestinal absorption were predicted. Additionally, toxicity risks in humans and drug-likeness were forecasted using Osiris property explorer software [131]. In this study, the authors, starting from certain docking calculations coupled with other in silico techniques, hypothesized the use of specific quinoline derivatives as targets for specific cancer cells. Exon-19 deletions such as E746_A750 (also known as Del19) and the L858R single-site mutation are among the major causes of NSCLCs. Resistance is caused by a secondary EGFR mutation, the T790M mutation. AZD9291 (osimertinib) is highly effective in treating EGFR-mutated NSCLCs with T790M-mediated drug resistance. To study the interaction between AZD9291 and EGFR kinase, Yan et al. (2020) performed unbiased MD simulations of the spontaneous binding of AZD9291 to L858R/T790M and to L858R [132]. Using MD simulation, the authors were able to explain the detailed interaction during dynamics of AZD9291 to EGFR mutants. Studies on natural compounds are promising to develop new anticancer drugs. Yu et al. (2020) illustrate how a natural compound, such as the flavonoid derivative formononetin, obstructs tumor development by inhibiting EGFR [133]. To identify natural products capable of inhibiting NSCLC cells, a comprehensive examination of 98 commercially accessible compounds was performed using MTS assay. Among these compounds, formononetin emerged as the most effective; thus, it was selected for computational studies. Yu et al. employed a molecular modeling technique to investigate the inhibitory activity of formononetin on WT and mutant EGFR. Homology modeling was performed to elucidate the three-dimensional configuration of the EGFR bearing exon 19 deletion mutant (residues E746–A750) on the crystal structure of wild-type (WT) EGFR (PDB: 4JR3) using MODELLER software [27,134]. The software generated ten models of the protein, which were assessed using the Discrete Optimized Protein Energy (DOPE) score implemented in MODELLER to select the most appropriate model for the docking calculations. Molecular docking findings indicated that the ligand successfully bound the WT and mutated proteins occupying the ATP-binding pocket, deeply penetrating the binding pocket and forming favorable hydrogen bonds. Molecular docking was performed using GLIDE (Schrödinger, 2013). The protein structures of WT and mutated EGFRs were retrieved from the PDB, and the exon 19 deletion EGFR structure was prepared using homology modeling. The proteins were prepared using the Protein Preparation Wizard in Schrödinger Suite 2013, whereas the ligand structure was prepared using LigPrep. The docking process was performed using the standard precision setting of Glide with default parameters. The docking poses were analyzed, and figures were generated using PyMOL [106]. Furthermore, the authors calculated the binding free energy using MM/GBSA calculations with the Prime MM/GBSA module in Schrödinger Suite 2013, with the general default settings and setting the residues with distances from the ligand within 12.0 Å. This paper, due to its extensive and complex in silico work on formononetin, provides evidence regarding how this natural compound has much potential as tumor suppressor. Lamellarin N, a natural marine-derived product, suppresses the activity of protein kinases that are associated with cancer and neurodegenerative diseases. Derivatives of Lamellarin N with A-ring modifications as non-covalent inhibitors of mutant EGFR have been reported. Fukuda et al. (2021) employed molecular docking in order to assess the interaction of a series of Azalamellarin N derivatives and the EGFR T790M/L858R mutant receptors [135]. Molecular docking simulations of the selected Azalamellarin N derivatives were performed after retrieving the crystal structure of the EGFR protein (PDB code: 4I22) using AutoDock, after the preparation of the ligand and EGFR pdb files using the AutoDock Tools program [39]. Gasteiger charges were calculated for ligands and proteins. A grid box measuring 24 × 22 × 22 Å^3^ with 1.000 Å grid spacing was used. Five docking runs were performed for each ligand, and the ligands were ranked based on their binding energy and binding affinity. The complexes of EGFR and the ligands were visualized and analyzed using PyMOL (version 2.3.4) [106]. In this study, the binding mode of zalamellarin N was found to be similar to that of Lamellarin N (1) in the ATP-binding pocket of EGFRs; with an additional hydrogen bond, the inhibitory activity was implemented. In this study, docking calculation was successfully used to predict the efficacy and potency of some inhibitors in the area of cancer disease. Joshi et al. (2021) employed molecular modeling methods to study benzamide-substituted chalcone derivatives as tyrosine kinase inhibitors targeting EGFRs and CDK2 [136]. Docking calculations were performed using Glide software (5.9) using wild-type and mutant EGFRs (T790M, T790M/L858R, and T790M/C797S) (PDB code: 1XKK) and CDK2 (PDB code: 2WXV) [137,138]. Desmond software was employed to perform MD simulation with an OPLS2005 force field [70,139]. This study indicates that, although the compounds bind to the mutant EGFR, the amino acid residues involved are similar to those of the wild-type EGFR. This observation suggests a limited selectivity, which opens up the modification of the substituted benzamide-calcon derivatives used here. Karnik at al. (2021) used in silico approaches to study novel substituted quinoline derivatives targeting the C797S mutated EFGR receptor [140]. Molecular docking calculations were performed using Glide to study binding affinity [141]. Proteins were downloaded from the PDB, prepared, and minimized, and the force field used was the OPLS3e [139]. After docking calculations, MD simulations were performed using the AMBER99 force field parameters for the duplex and the OPLS2005 force field for the ligands [67,68,139]. The combined docking/MD approach employed in this study is expected to be valuable for the future design, refinement, and development of potent EGFR inhibitors based on novel substituted quinoline derivatives. The research conducted by Khattab et al. (2021) focused on the development and synthesis of thienopyrimidine conjugates featuring a 1,2,3-triazole core and various sugar molecules through Cu(I)-catalyzed dipolar click cycloaddition [142]. The aim of this study was to inhibit the EGFR mutant receptor displaying the L858R mutation. Initially, the compounds were screened in vitro for their ability to inhibit EGFRs. After identifying the most effective ligands, they were further evaluated using molecular docking experiments. Specifically, the binding of compounds 2, 5, 7, and 13–18 to the EGFR ATP-binding pocket was studied using MOE software version 2008 [48]. The crystal structure of the EGFR bound to its inhibitor, gefitinib (PDB ID: 3UG2) [143], was obtained from the PDB, and the structure of the EGFR enzyme was prepared using the Protonate 3D protocol in MOE with default options. The docking analysis was carried out using MOE with the MMFF94x force field and partial charges were automatically calculated. The minimization process was performed with an RMSD gradient of 0.05 kcal.mol-1 Å-1 before docking. Initially, redocking was carried out for the gefitinib compound, which demonstrated near-perfect structural similarity, with an RMSD of 0.87 Å. The study of compounds 2, 5, 7, and 13–18 showed promising results for all the compounds, exhibiting energy scores ranging from 7.30 to 9.20 kcal/mol. Furthermore, these compounds displayed interactions with the Met793 residue, which was determined to be critical for the interaction with gefitinib. Additionally, the compounds formed several hydrogen bonds with their side chains, further enhancing their efficiency. The findings of the docking study and the in vitro experiments on EGFR indicated that the triazoles, oxymethyls, and β-D-glycosides play a crucial role in the extension of the chain and the improvement of the inhibitory effects of the compounds. All these results were used in the docking calculation to better understand the selectivity of a new class of thienopyrimidine conjugates with respect to EGFRs, highlighting their better behavior and potency with respect to classical doxorubicin and its derivatives.

Agarwal et al. (2022) aimed to identify novel inhibitors that could target the T790M/L858R (TMLR) mutations in the EFGR, which is a critical therapeutic target for overcoming drug resistance in lung cancer treatment [144]. In total, 150,000 molecules from different natural product libraries were screened using various ligand- and structure-based techniques. The crystal structure of 5EDQ with a co-crystallized ligand (5N3) was selected for virtual screening and molecular dynamics studies. The gap between positions 860–875 was filled using homology modeling, using the sequence ranging from 697 to 997 as the query, and the 3D structure of the double mutant (T790M/L858R) EGFR kinase as the template. MODELLER was employed to construct ten homology models, and the discrete optimized protein energy (DOPE) score was applied to determine the most suitable model [27,145]. Subsequently, FlexX-Pharm was used to screen the library of molecules predicted to possess anticancer activity in the previous step [146]. The chosen binding pocket consisted of the same amino acid that binds the co-crystallized inhibitor molecule 5N3, consisting of amino acid residues Leu718, Gly719, Phe723, Val726, Ala743, Lys745, Glu762, Leu788 Met790, Gln791, Leu792, Met793, Phe795, Gly796, Cys797, Asn842, Leu844, and Thr854. After docking, the Desmond molecular dynamics simulation software included in the Schrödinger Suite was used to perform 100 ns MD simulations using the OPLS 2005 force field [70,139]. Preparation of the system was performed using the TIP3P water model and neutralization of the system was performed by the addition of Cl- ions and the ionic strength of the solution was adjusted to 0.15 M by the addition of NaCl. The analysis of the graphs concerning the root mean square fluctuation (RMSF), solvent accessible surface area (SASA), radius of gyration (Rg), and root mean square deviation (RMSD) was conducted to examine the stability of the ligand and the conformational changes in the protein. Agarwal, in this paper, identified some ADMET adherent from natural products with the ability to potentially inhibit the double-mutated drug-resistant EGFR. Akher et al. (2022) employed docking and molecular dynamics simulations to study a novel covalent EGFR inhibitor to understand its effect on lung, breast, gastric, head, and neck cancers [147]. Docking calculations were performed using the UCSF Chimera Graphical User Interface. A non-covalent docking simulation was performed using AutoDock Vina [39]. The MD simulation was performed for 400 ns with a 2 fs time step. Molecular modeling techniques were employed in this study to elucidate the mechanism for the Cys797 alkylation reaction by Chlorofluoroacetamide-Based Covalent Inhibitors. The investigation of this activity on both the wild-type and mutated receptors thus presents the potential for developing novel drugs that can overcome EGFR resistance. Wu et al. (2022) investigated the effects of EAI0 45 and JBJ-04-125-02 as EGFR inhibitors using in silico methods [148]. The mutant-selective allosteric EGFR inhibitor EAI045 showed greater potency against EGFR-L858R and T790M compared to the WT; it was also effective against the C797S mutation. However, it was not effective as a single agent and required the co-administration of the anti-EGFR antibody cetuximab. A more potent EGFR inhibitor, JBJ-04-125-02, was developed. Molecular dynamics simulations were used to understand this mechanism. The X-ray crystal structures of EAI045 and JBJ-04-125-02 in a complex with EGFR (T790M) (PDB codes 6P1L and 6DUK) obtained from the PDB were used as the basis for constructing all simulation setups [149,150]. Protein structures were prepared and minimized using the Schrodinger 2017 (New York, NY, USA) Protein Preparation Wizard [119]. The Prime module was employed to add hydrogens and missing side chains. Epik was used to generate the protonation state of the side chain at pH 7 [82]. The Optimized Potentials for Liquid Simulations (OPLS) 2005 forcefield was employed for protein minimization [139]. Ligands were prepared using the Schrödinger suite Ligprep module, and mutant forms of the EGFR (L858R and T790M) were obtained by manually modifying the desired amino acid residues [151]. MD simulations were performed using AMBER 12 [67,68]. Amber forcefields were used in this study, in particular GAFF for EAI045 and JBJ- 04-125 02 and the AMBER 99SB force field for proteins [47]. The MD simulations were performed for 150 ns at 300 K with 1.0 atm pressure. The authors used MD here to provide evidence for the inhibitory mechanism of JBJ-04-125-02 as an EGFR inhibitor with the aim of finding new EGFR allosteric inhibitors. Saini et al. (2023) employed molecular modeling techniques to study novel compounds with anticancer properties targeting the EGFR [152]. In this study, 6524 compounds derived from Streptomyces sp. were subjected to drug-likeness filters, molecular docking, and a molecular dynamics simulation for 1000 ns to identify novel triple mutant EGFRCSTMLR (EGFR L858R/T790M/C797S) inhibitors. Molecular docking calculations were performed using the Glide software, in order to explore the binding modes and interactions of these compounds [141]. Consequently, MD simulations were performed using Desmond from Schrödinger during 1000 ns [70]. This computational study represents a promising strategy for the design of novel EGFR inhibitors with potent anticancer properties. The study identified compound C_42, derived from Streptomyces, as a potential natural inhibitor of the triple mutant EGFRCSTMLR for cancer therapy, showing superior binding affinity and stability compared to existing drugs like Osimertinib, with the benefit of being naturally sourced and potentially easier to produce through fermentation. In the study of novel ligands associated with wild-type and mutated proteins, virtual screening has been used to skim libraries of compounds and to identify those with the best characteristics, choosing from those with the best scoring values and the best interactions with the receptor. Todsaporn et al. (2023) used molecular modeling techniques to identify the most effective ligand from a library of compounds that exhibited activity against the wild-type EGFR and mutated EGFR [153]. This study aimed to identify ligands for the receptor target EGFR, a receptor tyrosine kinase whose mutations are associated with the onset of NSCLC and other types of tumors, such as breast cancer and glioblastoma multiforme. The receptor plays a key role in cell proliferation, angiogenesis, and apoptosis, and mutations in EGF receptors allow escape from negative regulation such as degradation [154]. EFGR receptors are single-chain transmembrane glycoproteins with an extracellular domain responsible for binding the ligand, a transmembrane domain, and an extracellular domain that contains the tyrosine kinase domain. They also used virtual screening to screen a library of 14 furo[2,3-c]pyridine derivatives. The structures of wild-type (PDB ID: 1M17), L858R/T790M (PDB ID: 4I22), and L858R/T790M/C797S EGFR (PDB ID: 6LUD) were obtained from the PDB, and the ligands were built and optimized using the Gaussian view 09 program [155,156,157,158,159,160]. Virtual screening and molecular docking studies were performed using GOLD software with a 10 Å sphere and 100 docking poses [159]. The docking results were visualized using the UCSD Chimera package [160] and the ligands were sorted based on their GOLD fitness score. For the molecular dynamics simulations, the structure of the complexes was determined using pmemd CUDA in AMBER 16 under periodic boundary conditions with the isobaric isothermal (NPT) ensemble in triplicate [67,68]. AMBER force fields were applied to the ligand (GAFF) and protein (FF14SB), and all the complexes were solvated using the TIP3P water model. The AMBER program LEaP was used to add missing hydrogens and minimize both ligand and protein. Molecular dynamics simulations were performed for 500 ns, and the RMSD was calculated. The binding free energies of the compounds were determined using the solvated interaction energy (SIE) method, and the binding free energy per residue decomposition was determined using the MM/PBSA of AMBER 16, based on the last 100 ns of the simulations. The molecular modeling results indicated that nine specific compounds possessed a superior VS score compared to the reference in both the wild-type (WT) and L858R/T790M double mutant EGFRs, and four compounds displayed better performance than the reference in the L858R/T790M/C797S mutant EGFR. The stability of the complex formed between the wild-type (WT) protein and the mutated protein with one of the top compounds, PD13, as well as the reference ligands was assessed using RMSD values from three replicates. The results of the binding energies per residue decomposition calculations indicated that both the wild-type EGFR/erlotinib and EGFR/PD13 compounds exhibited a similar binding mode. The analysis also highlighted that residue M793 is crucial for ligand interactions in mutated EGFR proteins, suggesting that the PD13 compound is a potential inhibitor of mutant EGFR. Despite numerous studies, many studies are still needed to identify a tyrosine kinase inhibitor that can overcome resistance due to receptor mutations in tumors involving EGFRs.

Somwar et al. (2020) studied gene fusions involving tropomyosin receptor kinases (TrkA/TrkC) encoded by the NTRK genes [161]. These act as oncogenic drivers across an array of cell lineages in both adult and pediatric solid and hematological malignancies. This involved the development of homology models leveraging the existing crystal structures of NTRK1 (TrkA) and NTRK3 (TrkC), which were subsequently coupled with meticulous MD simulations and molecular docking studies. The research was conducted on the V573M, F589L, G667C, and G67S mutations, using the type II NTRK inhibitor, alitretinoin, as a model to explore the resistance of mutations to the type I NTRK inhibitor, Larotrectinib [162]. The structure of the receptor was constructed employing the X-ray structures of the kinase domains of high-affinity NTRK1 (TrkA, PDB code: 4AOJ) and NTRK3 (TrkC, PDB code: 6KZC) using BLAST [25,163,164]. The PROCHECK module of the PDBSum server was employed to verify the stereochemical properties of the homology models, while the VERIFY3D module was employed to confirm the compatibility of the atomic models with their corresponding primary amino acid sequences [116,165]. This integrative approach provides a platform to dissect the conformational dynamics within the NTRK1 kinase domain and discern the disparate sensitivity of mutations to two pivotal inhibitors, larotrectinib and altiratinib. Docking studies revealed a pronounced seven-fold decrease in the affinity of larotrectinib towards the active form of NTRK1V573M compared to wild-type NTRK1. This diminished binding propensity was attributed to steric clashes engendered by larotrectinib, which encountered the thioether moiety within M573. Conversely, altiratinib exhibited a reduced IC50 for NTRK1V573M (2.8 nM) relative to NTRK1wt, suggesting a potentially distinct binding modality. Further insights obtained from dissociation constant calculations underscored a four-fold reduction in the affinity of altiratinib for NTRK1V573M compared to NTRK1wt, consistent with empirical observations from cell-based assays. Structural modeling suggested that this diminished binding might be attributed to the stabilizing influence of S/π interactions between the thioether moiety in methionine and the aromatic ring within the structural framework of altiratinib. These findings provide invaluable insights into the intricate molecular mechanisms underlying the variable responsiveness of NTRK1 mutations to specific inhibitors, thereby catalyzing the refinement of targeted and efficacious therapeutic modalities tailored for cancers propelled by NTRK gene fusion.

Fibroblast growth factor receptors (FGFRs) are among the tyrosine kinase receptors that are important for their involvement in various types of cancer. The dysregulation of FGFRs has been implicated in ovarian carcinoma and lung adenocarcinoma. Several ligands targeting FGFR receptors are currently under evaluation for the treatment of various types of cancer [166]. Dehghanian et al. (2021) studied the effects of LY2874455 (or 6LF), a pan-FGFR inhibitor, identified as the most efficient TKI for all resistant mutations in FGFRs [167]. Four FGFR4 systems were prepared for MD simulations using Amber18 [67,68]. During the preparation of the system, the missing hydrogens were added, the system was neutralized, and all the complexes were solvated in an octahedral TIP3P water model box. The system was then gradually warmed from 0 to 310 K over a period of 500 ps, followed by a 1 ns equilibration period. Finally, all the systems were subjected to production MD simulations for 200 ns, and after the simulations, trajectory analyses were performed using CPPTRAJ and R package Bio3D 2.432 [106,168]. The overall conformational changes in all the trajectories were confirmed by evaluating the pairwise RMSD of the backbone using the Bio3D package. Molecular visualizations were created using PyMOL 2.4, and 310 plots were created using the ggplot2 package. MD simulations in this case perfectly show that the pan-FGFR inhibitor 6LF can bind efficiently to both the WT and V550L FGFR mutants, which will allow for developing more efficient TKIs as targets of the FGFR signaling pathway. This study showed that LY2874455 was effective in inhibiting FGFR4 mutated receptor activity, particularly the N535K mutation, which is associated with resistance to other FGFR inhibitors, suggesting that it could be a promising candidate for the treatment of tumors with FGFR4 mutation-mediated resistance.

ROS-1 proteins have a role in signaling pathways involved in cell growth, differentiation, and proliferation. In particular, mutations in ROS-1 proteins have been observed in lung cancer. For this reason, several studies have focused on the design of ligand that can target ROS-1 proteins [169]. Pathak et al. (2021) employed molecular modeling calculations to investigate a series of benzimidazol-2-amine derivatives based on pharmacophore studies of ROS-1 [170]. The study focused on two proteins: WT ROS-1 and the Gly2032Arg mutant ROS-1. Discovery Studio software (4.1) was employed to generate the receptor–ligand pharmacophore model [69]. Then, Glide software from Schrödinger was used to perform docking calculations. Desmond from Schrödinger was used to perform MD simulations of 30 ns, with the OPLS2005 force field [139]. This study reports an interesting screening of a database of small molecule heterocycles adopting a double approach of docking and MD to evaluate the stability of the complexes.

FMS-like tyrosine kinase 3 (FLT3) are tyrosine kinase receptors involved in hematopoietic cell growth, proliferation, and differentiation. Consequently, these receptors are implicated in acute myeloid leukemia. Although FLT3 inhibitors have been identified, several resistant mutations have recently been observed [171]. Wang et al. (2022), studied FLT3 mutations ASP-835 and (D835F/H/V/Y), which are responsible for resistance in the treatment of acute myeloid leukemia using in silico approaches [172]. The crystal structures of WT FLT3 in their inactive and active conformations were retrieved from the PDB. chimer from Schrödinger was used for the construction of the initial complex. All complex structures were used for subsequent MD simulations. MD simulations of 100 ns were performed using the OPLS2005 force field [139]. The study showed that the A-loop of the FLT3 protein exhibits conformational changes due to the resistant mutation at Phe830. The analysis of protein–inhibitor interactions revealed that movements of this amino acid can affect the binding of sorafenib, but not crenolanib. This work suggests that the structural impact of the D835V mutation should be considered when designing novel therapeutic agents targeting FLT3.

Kuznetsov et al. (2020) focused on the molecular mechanisms underlying the activation of the insulin receptor (IR) and other proteins in its family that remain elusive [173]. Molecular dynamics simulations of the IR ectodomain revealed significant collective motions that may be responsible for the closure of the C-terminus of the FnIII-3 domains and the spatial approximation of the TM helices during insulin-induced receptor activation. A single functional mutation in the TM domain has been described for each receptor, V938D in IR and V912E in IGF-1R. MD simulations performed using Gromacs software version 5.1 demonstrated that both induce basal receptor activity [63]. Simulations were performed for a duration of 150 ns with constraints placed on the backbones of L1, CR, L2, and FnIII-1,2,3 and subsequently for a duration of 200 ns without constraints. Following the MD simulations, the ECD motion of IR was studied using principal component analysis (PCA). Two PDB files were used for the analyses, one representing the basal structure (PDB code: 4ZXB) and the other representing the protein I in the active state (PDB code: 6SOF) [174,175]. All carbohydrate/antibody fragments and reconstructed missing regions were excluded. Then, to equilibrate conformations of insert domains (IDs) and interdomain loops, 150 ns MD trajectories with restraints imposed on backbone atoms of the rigid domains L1, CR, L2, and FnIII-1,2,3 were calculated. Finally, 200 ns long trajectories were obtained without constraints. In this case, molecular modeling techniques have made it possible to understand how the insulin receptor (IR) is activated by the presence of the ligand, emphasizing in particular the amino acid residues involved in wild-type and mutated receptors.

### 3.3. Ion Channel Receptors

Ion channel receptors are transmembrane proteins that form channels extending across cellular membranes. These receptors open and close in response to various stimuli, allowing ions to cross the membrane in either direction [176].

Transient receptor potential (TRP) channels are receptors responsive to several ligands and involved in multiple signaling pathways. Vanilloid TRP (TRPV) channels exhibit sensitivity to vanillin and capsaicin and participate as sensors of pain sensations [177]. The effects of mutations in the TRPV1 receptor on pain stimuli remain poorly understood. Lubova et al. (2020) used molecular modeling to study the effect of the TRPV1 point mutants G643A, I679A + A680G, and K688G/P [178]. Four MD simulations of the mutant receptor were performed to estimate the effect of replacing Gly 643 with Ala, Ile 679/Ala 680 with Ala/Gly, and Lys 688 with Gly and Pro, and the results were compared with those obtained for the wild-type receptor. In silico mutagenesis was performed in PyMOL using either the open or closed state of the receptor (residues 427–719) [106]. The structure was inserted into a pre-equilibrated lipid bilayer consisting of palmitoyloleoylphosphatidylcholine (POPC), palmitoyloleoylphosphatidylethanolamine (POPE), and cholesterol (CHOL) molecules [179,180]. MD simulations were performed using the GROMACS 2019 package and Amber99sb-ildnforce field, and a twin-range (10/12 Å) spherical cutoff function was used to truncate the van der Waals interactions [63]. MD simulations were carried out in an isothermal (NPT) ensemble with a semi-isotropic pressure of 1 bar and a constant temperature. An analysis of the MD trajectories was performed using utilities from the GROMACS package. In this case, the computational approach was used to identify the possible residues that affect the activation and deactivation of TRPV1 and, as a consequence, the amino acid mutations inside receptors involved in nociception.

The purinergic receptors (P2X4R) are ion-gated channels responsive to adenosine triphosphate (ATP) [181]. These receptors are widely distributed in CNS and immune cells, and they participate in several important functions. Popova et al. (2020) in their study attempted to investigate alcohol use disorder (AUD) [182]. Since ethanol acts on purinergic P2X4R receptors, they found that arginine at position 33 (R33) in the TM1 segment plays a role in the ethanol sensitivity of these receptors [183]. The structure was studied by constructing two new homology models of rat P2X4 using DISCOVERY STUDIO 3.5 (DS 3.5; Biovia, San Diego, CA, USA) and two structures (open and closed) of P2X4 from zebrafish [69]. The software was used to generate 50 models. For these models, the side chain rotamers were improved, and the backbone atoms remained fixed using the Biovia version of the CHARMM force field within DS 3.5. This paper highlights new residues switching ethanol activity on P2X4Rs, providing new insight for the development of new AUD drugs.

The study by Katz et al. (2021) aimed to investigate the role of nicotinic acetylcholine receptors (nAChRs) in several disorders [184]. Specifically, researchers examined the potential of α-conotoxins (α-CTX) as a naturally selective and competitive antagonist of nAChRs. These antagonists have shown great promise in the treatment of nAChR disorders. Homology modeling was employed to create the structures of the two receptor subtypes, α3β2 and α3β4. Subsequently, free energy perturbation (FEP) techniques were applied, and the results were compared with MM/GBSA calculations to investigate the influence of 11 mutations on the interaction with α-CTXs. Homology models were constructed using the “build homology model” function in Maestro using the crystal structure of protein downloaded from the PDB (PDB code 5XGL) as a template. MD simulations were performed using Desmond software [70]. The 19th frame for the α3β2 nAChR and the 1st frame for the α3β4 nAChR were then applied as inputs for FEP and MM/GBSA calculations. MM/GBSA calculations were performed using default settings for all the residues. FEP calculations were performed using the same input structure as the MM/GBSA calculations using default settings. The results of the calculations show that the FEP method demonstrated a notable ability to predict which mutation can improve the selectivity or potency of α-CTXs for nAChRs. In contrast, the MM/GBSA method did not demonstrate this ability.

### 3.4. Nuclear Receptors

Nuclear receptors function as transcription factors activated by hormones or steroids [185]. Androgen receptors (ARs) are nuclear receptors that mediate the functions of androgens such as testosterone. These receptors are distributed in numerous tissues and exhibit several biological activities, depending on their localization [186]. The most significant biological activity is associated with the male reproductive system. Mutations in these receptors are associated with disorders of sex development [187]. Antiandrogen medications have been developed to address several pathologies, including benign prostatic hyperplasia, prostatic cancer, acne, and androgenetic alopecia. Shao et al. (2021), performed docking calculations and molecular dynamics simulations of the existing marketed antiandrogens with the wild-type and F876L mutant ARs to understand the effect of the mutation on the binding with the ligands [188]. Docking calculations using both eild-type and mutated receptors were performed with Glide software [141]. The F876L mutant was constructed based on the docked complex structure of the wild-type AR. MD simulations were performed using pmemd.cuda in AMBER18 with the ff14SB force field [67,68]. This paper used docking/MD calculation to successfully study residue-specific binding. Information obtained will be highly valuable for understanding the mechanism of drug resistance related to specific mutations and for designing next-generation drugs to combat potential new mutations.

In the field of cancer, several studies were performed to investigate the role of the estrogen receptor (ER). These receptors are activated by estrogens, and they are involved in cell proliferation and differentiation, therefore crucial for cancer. In particular, they are involved in breast, ovarian, and endometrial cancers [189]. A challenge in the treatment of these tumor types is precisely that the mutated receptor lacks sensitivity for certain inhibitors used in therapy. This has prompted research in the direction of deepening the study of these receptors in order to identify active ligands. Chinnasamy et al. (2020) used molecular modeling techniques to study the activity of Elacestrant (ELA), a new class of selective estrogen receptor down-regulators (SERDs) used for the treatment of cancer, with a particular focus on the L536S mutated receptor [190]. They performed molecular docking and MD simulations on both wild-type and L536S mutant ERα to understand the mechanics of the interaction between the receptor and the novel ligand. Molecular dynamics simulations were performed using AMBER with a duration of 100 ns to study the difference in the antagonism of the ELA compound. Gaussian03 was used to perform the structure minimization [158]. Consequently, the ligand preparation and minimization was performed using the OPLS2005 force field after generating the different ionization states of the ELA molecule using Epik state penalties [139,151]. The protein structure was downloaded from the PDB, and the missing side chains or residues were added using the Prime routine [119]. The L536S mutant was manually generated from the wild type Erα structure using PyMOL [106]. The Glide module in Maestro from Schrödinger was used to perform flexible docking [51]. The system was placed in a TIP3P water box and then neutralized with Na+ ions. The trajectories obtained from the MD simulation were analyzed using VMD to understand the conformation and stability of the system [102]. In this work, other techniques apart from docking and MD were adopted, such as the study of the binding free energy using MM/GBSA methods and PCA to study the inhibition mechanism and down-regulation of the ERα-ELA complex. Mayne et al. (2021) investigated how single point mutations in the estrogenic receptor led to drug therapy failure [191]. The authors employed molecular dynamics simulations using Nanoscale Molecular Dynamics (NAMD) software and free energy calculations using the BEUS method [64]. They discovered that, over time, patients on long-term therapy stop responding to treatment, causing the disease to become metastatic. This happens because mutations in the ligand-binding domain (LBD) of the ER enable the receptor to activate without binding to its ligand [192,193]. The study highlighted two particularly critical mutations: Y537S and D538G, which were found to be the primary drivers behind this drug resistance and disease progression. In the field of research on ERs, many authors recognize the Y537S mutation in estrogenic receptor α (ERα) as a notable mutation [194]. Shylaia et al. (2021) investigated the efficacy of coumarins against the Y537S mutation with the aim of identifying the key chemical features necessary to inhibit its activity [195]. Docking studies were conducted using Glide from Schrödinger suite version 2009, LLC, New York, for coumarin compounds to investigate their interactions with active site residues. The compounds were prepared using LigPrep from Glide and then the docking calculations were performed. This approach not only involved evaluating binding energies within the active domain, but also delved into MD simulations using the Desmond module [70]. First, a system was built by adding a water model and neutralizing the system. Subsequently, minimization was performed. Finally, MD simulations were conducted for 150 ns. These simulations provided insights into how ligands induce allosteric signals, ultimately influencing the balance between the active and inactive conformers of the Y537S ERα. Understanding these dynamics was crucial for discerning the agonistic or antagonistic nature of coumarin compounds, thereby informing the development of potential therapeutic strategies for ERα-positive breast cancer.

### 3.5. Other Receptors

The urokinase-type plasminogen activator receptor (uPAR) is a protease binding receptor involved in the regulation of inflammation, cancer, and other pathological conditions. Xu et al. (2021) employed molecular modeling techniques to study the interaction of several uPAR inhibitors derivative from the protein–protein uPAR H47C-N259C mutant interface [196]. In particular, the authors performed docking and molecular dynamics simulations. The structure of Upar-Upa-vtn obtained from the PDB (PDB ID: 3BT1) was prepared using Protein Preparation Wizard in Schrodinger (version 2018). The structures underwent a protonation process at pH 7 and the ligands were prepared using LigPrep of Epik [151]. Docking was performed using the tool “Ligand Docking” and “Induced fit docking” using Glide in Schrodinger with the default options for docking [51]. MD simulations were performed using the AMBER16 software package [67,68]. The complexes of the proteins with the ligands were placed in a water box and neutralized with Na+ or Cl- counterions. Ff14SB, gaff, and TIP3P force fields were used. MD simulations were performed with a duration of 200 ns per simulation for each protein–compound complex. This study suggests that effective inhibition of uPAR binding to uPA with small molecules will likely require the development of compounds that exhibit high affinity for the solution apo structures of uPAR, rather than the uPA-bound forms of the receptor.

### 3.6. Enzymes

Enzymes are biomolecules that function as catalysts for biological reactions. Their role is to accelerate biochemical reactions, and they perform this acceleration because they lower the activation energy for reactions. They bind to specific targets and, consequently, catalyze specific reactions. Enzymes participate in a wide range of biological process [197]. Lipoxygenases are enzymes that catalyze the oxygenation of fatty acids. The reaction leads to the formation of hydroperoxides from polyunsaturated fatty acids [198]. Since the structure of lipoxygenase is unknown, Tsai et al. (2021) constructed a dimer model using a combination of computational methods, experimental mutagenesis, mutagenesis, and hydrogen–deuterium exchange (HDX) studies [199]. The homology model of h12-LOX was constructed using PRIME (version 4.7, Schrödinger Inc., New York, NY, USA) [119]. The h12-LOX model was then energy-minimized using Protein Preparation Wizard (Schrödinger Inc.). Site-directed mutagenesis was used to generate the lipoxygenase structure. Leu183 and Leu187 were replaced with negatively charged glutamate residues, and the adjacent aromatic residues were replaced with alanine residues (F174A/W176A/L183E/L187E/Y191A). The PDB structure of h12-LOX with code 3D3L was missing many residues, such as helix alpha11 [200]. This work highlights useful aspects when it comes to better understanding the stability of h12-LOX, as well as biochemical properties that open up possibilities for future drug design studies.

### 3.7. Other Proteins

Affitins are small artificial proteins that have a role in therapeutic and diagnostic fields as they can be used as antibody substitutes. Loussouarn et al. (2020) in their study described the stability of affitins under simulated conditions of gastric and intestinal digestion [201]. They appear to be degraded into stable fragments in the gastric medium in vitro. Pepsin-generated cleavage sites were identified and silenced by site-directed mutagenesis. Three mutants, M1 (F6W-F7I), M2 (L31I-F32W), and M3 (L54I-L55V-L58I), were designed to evaluate the effect of the mutated amino acids on proteolytic degradation by pepsin [202]. Homology modeling was performed using the crystal structure of the H4 afftin with lysozyme and PyMOL software to visualize the effect of the mutations [106]. Here, homology modeling was used to study the stability of afftin at various pHs and temperatures with the aim of engineering instruments.

Dachshund homolog 1 (DACH1) is a protein involved in the regulation of tumorigenesis. In particular, this protein exhibits tumor-suppressive functions, significant in both tumor growth and metastasis [203]. Hassan et al. (2022) focused their work on the structural and mutational analysis of DACH1 in relation to carcinogenesis [204]. Mutations in DACH1 are associated with tumor progression and suppression. DACH1 has been highlighted as a molecular target for successful prognosis and therapeutics in carcinogenesis. The crystal structure of DACH1 is not available in the PDB. Therefore, researchers have used homology models to predict wild-type and mutant (C188Y) DACH1 models. Using the SWISS MODEL approach, eight different templates from the library were identified, and high-quality templates were then selected for modeling DACH1 [26]. The models were built based on target-template alignment using ProMod3 [160]. The dynamics simulations were performed using GROMACS 5.1.4 (Groningen Machine for Chemical Simulations) in explicit water [63]. MOE and VMD were used for the visualization and molecular inspection of proteins and ligands [48,102]. Subsequently, energy minimization for the system was performed, which was followed by equilibration of the system using two sequential NVT (100 ps) and NPT (100 ps) runs, during which the heavy atoms of the protein were restrained. After minimization, the resulting ensembles were subjected to a 100 ns MD simulation. The trajectories were stored at 5 ns intervals for the analysis. The root mean square deviation (RMSD) of the protein (backbone and side chains), root mean square fluctuation (RMSF), and radius of gyration (Rg) of amino acids were determined [205,206]. The structures of the model (C188Y) and wild-type DACH1 protein residues were plotted using XMGRACE. The work of Hassan and colleagues concluded that DACH1 could serve as a promising therapeutic target for the prognosis and treatment of carcinoma development.

Integrins are integral membrane proteins that function as cell surface receptors involved in cell signaling. Their role in cell signaling is significant in angiogenesis and, consequently, in cancer. Bhattacharya et al. (2020) used an in silico molecular modeling approach to investigate the effect of the deletion of the pentapeptide (PPQEE) on the structure and conformation of the integrin αV subunit in detail [207]. The integrin αV subunit sequence was subjected to BLAST analysis [25]. The X-ray crystal structure of integrin αV in PDB ID showed that 4G1E contained the 100% sequence but did not cover the cytoplasmic domain region of the protein subunit [208]. Therefore, molecular modeling techniques were used to model the complete structure of the wild-type (WT) integrin αV subunit. Homology modeling was performed using SWISS MODEL [26]. Therefore, the complete models of the WT and MT integrin αV subunits were generated using the RaptorX server [209]. RaptorX generated models for both the WT and MT integrin αV subunits that covered 100% of the residues. This study highlights how proteins’ native structure is greatly affected by PPQEE residues.

### 3.8. Considerations

Numerous in silico approaches have been used extensively in the studies described, proving their fundamental role in deepening our understanding of the role of receptor mutations in disease onset and mechanisms of treatment resistance. These computational tools have proven particularly effective in the study of GPCRs and tyrosine kinase receptors, which are significant elements in the development of several types of cancer. Through the use of advanced molecular modelling techniques, it has been possible to identify specific mutations responsible for drug resistance and to design novel effective inhibitors. These methods represent valuable resources for the development of innovative and personalized therapies. The molecular modeling methods and software employed in all the studies cited in this review are summarized in Table 1.

## 4. Conclusions

The number of recent studies involving in silico approaches in life sciences is rapidly increasing, both in quantity and in the variety of new techniques. In this review, mutations in receptors associated with human pathologies, both rare and common, were considered. The majority of these studies employed docking and molecular dynamics simulations, which have emerged as the most frequently employed in silico approaches. Moreover, the studies reported here show that in most cases, a more comprehensive analysis was performed by coupling more than one computational technique. We also noticed that some new approaches, such as Threading or Electron Density Analysis, and the highly challenging AlphaFold algorithm, might become crucial in drug discovery. A closer look at recent studies on receptor mutations involved in human pathologies underscores the continued importance of cancer research, although these studies could easily be extended to other significant pathologies and rare diseases. Moreover, while docking calculations have been successfully used to predict new ligands for specific receptors or for facilitating rapid drug design, homology modeling appears to be an ancillary technique. In the cases studied here, it helps when it comes to understanding certain properties and mechanisms but does not offer direct and quick applications. Molecular dynamics, a powerful yet slow method, has been effectively coupled with docking to serve as an efficient tool for drug design. Additionally, virtual screening, while needing support, also provides substantial assistance in preliminary studies. When combined with docking calculations, research has gained significant momentum in the discovery of new potent molecules.

## Figures and Tables

**Table 1:** Summary of molecular modeling techniques and software employed in reviewed studies.

Author	Target	Receptor	Disease	Molecular Modeling Technique	Software
Zariñán et al. (2021) [83]	FSH	GPCRs	Cancer	Docking, MD	MOE, GROMACS [49,63]
De Filippo et al. (2020) [87]	A2AR	GPCRs	Cancer	Docking, MD	MOE, NAMD [49,64]
Wang et al. (2020) [91]	A2AR	GPCRs	Cancer	Homology modeling	GPCR-ModSim [92]
Bony et al. (2022) [95]	GABA B	GPCRs	Pain	Docking, MD	AutoDock Vina, GROMAC S [29,54,63,100]
Casajuana-Martin et al. (2022) [104]	CB2	GPCRs	Pain	MD	GROMACS [63]
del Torrent et al. (2023) [107]	CB1R, CB2R	GPCRs	Pain	MD	GROMACS [63]
Chan et al. (2020) [109]	MT1, MT2	GPCRs	Alzheimer’s, Parkinson’s, depression	Homology modeling, Docking	SWISS MODEL, AutoDock Vina, SITEHOUND, FunFOLD [26,39,54,100,112,113]
Rodriguez-Contreras et al. (2021) [114]	D2	GPCRs	Hyperkinetic movement disorder	Homology modeling, MD	VERIFY3D, YASARA [115,116]
Picarazzi et al. (2022) [117]	rhodopsin	GPCRs	Rertinis pigmentosa	Homology modeling, MD	FASTA, Prime [119]
Akher et al. (2020) [125]	EGFR	TK receptors	Cancer	MD, MMGBSA	AMBER [68]
Jingwen et al. (2020) [126]	EGFR	TK receptors	Cancer	Docking, MD	AutoDock Vina, GROMAC S [39,54,63,100]
Shaheen et al. (2020) [128]	EGFR	TK receptors	Cancer	Docking, ADME	MOE, PreADME [49,130]
Yan et al. (2020) [132]	EGFR	TK receptors	Cancer	MD	Desmond [70]
Yu et al. (2020) [133]	EGFR	TK receptors	Cancer	Homology modeling, Docking, MM/GBSA	MODELLER, Glide, Prime [27,51,119]
Fukuda et al. (2021) [135]	EGFR	TK receptors	Cancer	Docking	AutoDock Vina [39,54,100]
Joshi et al. (2021) [136]	EGFR	TK receptors	Cancer	Docking	Glide [51]
Karnik at al. (2021) [140]	EGFR	TK receptors	Cancer	Docking, MD	Glide [51]
Khattab et al. (2021) [142]	EGFR	TK receptors	Cancer	Docking	MOE [49]
Agarwal et al. (2022) [144]	EGFR	TK receptors	Cancer	Homology modeling, Docking, MD	MODELLER, Desmond [27,70]
Akher et al. (2022) [147]	EGFR	TK receptors	Cancer	Docking, MD	UCSF Chimera, AutoDock Vina [39,54,100,160]
Wu et al. (2022) [148]	EGFR	TK receptors	Cancer	MD	AMBER [68]
Saini et al. (2023) [152]	EGFR	TK receptors	Cancer	Docking, MD	Glide, Desmond [51]
Todsaporn et al. (2023) [153]	EGFR	TK receptors	Cancer	VS, MD, MM/GBSA	Gold, AMBER [68]
Somwar et al. (2020) [161]	NTRK	TK receptors	Cancer	Homology modeling	BLAST [25]
Dehghanian et al. (2021) [167]	FGFRs	TK receptors	Cancer	MD	AMBER [68]
Pathak et al. (2021) [170]	ROS-1	TK receptors	Cancer	Pharmacophore model, Docking, MD	Discovery Studio, Glide, Desmond [51,69,70]
Wang et al. (2022) [172]	FLT3	TK receptors	Cancer	Docking, MD	Glide, Desmond [51,70]
Kuznetsov et al. (2020) [173]	IR	TK receptors	Cancer	MD	GROMACS [63]
Lubova et al. (2020) [178]	TRPV1	Ion channel receptors	Pain	MD	GROMACS [63]
Popova et al. (2020) [182]	P2X4R	Ion channel receptors	Alcohol use disorder	Homology modeling	Discovery Studio [69]
Katz et al. (2021) [184]	nAChRs	Ion channel receptors	Pain	Homology modeling, MD, MM/GBSA, FEP	Maestro, Desmond [70]
Shao et al. (2021) [188]	AR	Nuclear receptor	Cancer	Docking, MD	Glide, AMBER [51,68]
Chinnasamy et al. (2020) [190]	ER	Nuclear receptor	Cancer	Docking, MD, MM/GBSA	Glide, AMBER [51,68]
Mayne et al. (2021) [191]	ER	Nuclear receptor	Cancer	Docking, MD	Glide, NAMD [51,64]
Shylaia et al. (2021) [195]	ER	Nuclear receptor	Cancer	Docking, MD	Glide, Desmond [51,70]
Xu et al. (2021) [196]	uPAR	Proteins	Cancer	Docking, MD	Glide, AMBER [51,68]
Tsai et al. (2021) [199]	h12-LOX	Enzyme	Inflammatory diseases	Homology modeling	Prime [119]
Loussouarn et al. (2020) [201]	Affitins	Proteins	Gastric and intestinal digestion	Homology modeling	Not disclosed
Hassan et al. (2022) [204]	DACH1	Proteins	Cancer	Homology modeling, docking, MD	SWISS MODEL, MOE, GROMACS [26,49,63]
Bhattacharya et al. (2020) [207]	integrin αV	Proteins	Cancer	Homology modeling	BLAST, SWISS MODEL [25,26]

## Data Availability

No new data were created or analyzed in this study.
